# Biomechanical properties of retina and choroid: a comprehensive review of techniques and translational relevance

**DOI:** 10.1038/s41433-021-01437-w

**Published:** 2021-03-01

**Authors:** Mariantonia Ferrara, Gaia Lugano, Maria Teresa Sandinha, Victoria R. Kearns, Brendan Geraghty, David H. W. Steel

**Affiliations:** 1 Newcastle Eye Centre, Royal Victoria Infirmary, Newcastle upon Tyne, UK; 2 Department of Eye and Vision Science, Institute of Life Course and Medical Sciences, University of Liverpool, Liverpool, UK; 3 St Paul’s Eye Unit, Royal Liverpool University Hospital, Liverpool, UK; 4 Musculoskeletal and Ageing Science, Institute of Life Course and Medical Sciences, University of Liverpool, Liverpool, UK; 5 Sunderland Eye Infirmary, Sunderland, UK; 6 Bioscience Institute, Newcastle University, Newcastle Upon Tyne, UK

**Keywords:** Biological techniques, Medical research

## Abstract

Studying the biomechanical properties of biological tissue is crucial to improve our understanding of disease pathogenesis. The biomechanical characteristics of the cornea, sclera and the optic nerve head have been well addressed with an extensive literature and an in-depth understanding of their significance whilst, in comparison, knowledge of the retina and choroid is relatively limited. Knowledge of these tissues is important not only to clarify the underlying pathogenesis of a wide variety of retinal and vitreoretinal diseases, including age-related macular degeneration, hereditary retinal dystrophies and vitreoretinal interface diseases but also to optimise the surgical handling of retinal tissues and, potentially, the design and properties of implantable retinal prostheses and subretinal therapies. Our aim with this article is to comprehensively review existing knowledge of the biomechanical properties of retina, internal limiting membrane (ILM) and the Bruch’s membrane–choroidal complex (BMCC), highlighting the potential implications for clinical and surgical practice. Prior to this we review the testing methodologies that have been used both in vitro, and those starting to be used in vivo to aid understanding of their results and significance.

## 摘要

研究生物组织的生物力学特性对于提高我们对疾病发病机制的理解至关重要。我们对角膜、巩膜和视神经的生物力学特征已经通过广泛发表的文献和学者们对其重要性的深入理解已经认识得很透彻, 而相比之下, 人们对视网膜和脉络膜的认知相对有限。了解这些组织不仅对于阐明各种视网膜和玻璃体视网膜疾病如老年黄斑变性、遗传性视网膜营养不良和玻璃体视网膜界面疾病等潜在的发病机制很重要, 而且对于优化视网膜组织的手术处理以及植入式视网膜假体和视网膜下治疗的设计和特质也很重要。本文旨在全面回顾视网膜、内界膜(ILM)和Bruch膜-脉络膜复合体(BMCC)的生物力学特性的现有知识, 强调其对临床和外科手术的潜在影响。在此之前, 我们回顾了已经用于体外和开始在体内使用的生物力学检测方法, 以帮助理解其结果和意义。

## Introduction

Diseases affecting the retina, including age-related macular degeneration (AMD) and hereditary retinal dystrophies, comprise a large proportion of untreatable blindness globally. Considerable efforts have been made into unravelling their underlying pathogenesis, but there has been relatively little study into the biomechanical properties of the ocular tissues affected during normal human development and ageing.

Biomechanics aims to characterise the origin and effects of mechanical forces involved in biological processes at different levels, from whole body/organ down to the subcellular level [1]. Soft biological tissues, including retina and choroid, can be regarded as hierarchical, collagenous structures exhibiting a complex biomechanical behaviour. This is directly related to their composition and microstructural organisation and can be described in terms of their age-dependent, anisotropic, non-linear (hyperelastic) and viscoelastic^1^* properties. Indeed, age-related changes in elastic and collagenous fibres resulting in the variations of biomechanical properties, may play a significant role in the pathophysiology of age-related ocular diseases, in particular AMD [2]. Similarly, changes can be linked to other ocular features, such as the increased stiffness of the retina–choroid–sclera complex as axial length increases [3, 4].

A more in-depth knowledge of the biomechanical properties of these tissues could improve our understanding of the pathogenic mechanisms of disease. For instance, it is known that tangential and anterior-posterior forces related to vitreous ageing are involved in the origin of vitreoretinal interface (VRI) diseases; [5] however, the exact effect of these processes is not fully understood. Moreover, many vitreoretinal diseases are currently treated with pars plana vitrectomy that involve direct manipulation of retinal tissue. A better understanding of retinal biomechanics might help us to identify safe force thresholds and/or optimal angles of membrane peeling and, thereby, improved surgical approaches to minimise trauma [6, 7]. Similarly, a more detailed knowledge of retinal anisotropic behaviour could result in optimisation of implantable retinal prostheses and advanced therapeutics, e.g. subretinal therapies [7].

There have recently been comprehensive reviews of the biomechanical properties of the sclera and optic nerve head (ONH) in the context of glaucoma and myopia in particular [6, 8–13]. This manuscript aims to review existing knowledge, and the techniques used to investigate the biomechanical properties of retina and the Bruch’s membrane/choroid complex (BMCC).

## Methods

The studies for this review were initially identified using Medline and Embase to December 2019, searching also the reference lists of the studies selected. The MeSH terms used were: ocular biomechanics; eye biomechanics; elasticity; stiffness; anisotropy; thickness; retina; choroid; Bruch’s membrane; internal limiting membrane; Young’s modulus; elasticity modulus; biomechanical tests. A subsequent search was also carried out using Scopus, also to December 2019, with the following search terms: retina, choroid, uniaxial, tensile, tension, compression, atomic force microscopy and optical coherence elastography. We have tabulated and summarised the most relevant publications in Table 1.

**Table 1:** Experimental studies investigating the biomechanical properties of retina, internal limiting membrane and/or choroid.

Author(s), reference	Eye model	Study of ageing or eye pathology (if applicable)	Tissue(s) analysed	Test used	Properties Measured	Main biomechanical findings
Moses [19]	Human		Choroid	Uniaxial tensile	Breaking force Tensile strength Young’s modulus Anisotropy	Meridional strips stiffer than equatorial strips
Deguillebon and Zauberman [26]	Rabbit	Ageing	Retina	Peeling	Strain rate	Increased stretching rate increases retina stiffness
Graebel and van Alphen [35]	Human	Ageing	Choroid	Uniaxial tensile	Young’s modulus	Stiffness increases with age
Wu et al. [20]	Cow, rabbit		Retina, choroid	Uniaxial tensile	Young’s modulus Anisotropy	The presence of blood vessels increases retinal stiffness
Friberg and Lace [21]	Human	Ageing	Choroid	Uniaxial tensile	Young’s modulus Anisotropy	Choroid is stiffer posteriorly to equator
During et al. [22]	Cow		Retina	Uniaxial tensile	Anisotropy	Strain in a retinal strip under tension is not uniform
Reichenbach et al. [78]	Rabbit	Ageing	Retina		Tensile strength Young’s modulus Anisotropy	Retinal tensile strength increases with age for all regions
van Alphen and Graebel [23]	Human	Ageing	Choroid	Uniaxial tensile	Young’s modulus Anisotropy	Meridional/equatorial strip stiffness similar but increases with age
Jones et al. [77]	Cow		Retina		Young’s modulus	Retina Young’s modulus: ~20 kPa
Donsey et al. [24]	Pig		Retina	Uniaxial tensile	Tensile strength Ultimate strain Anisotropy	Meridional strips endure higher stresses than equatorial strips
Wollensak et al. [32]	Pig		Retina	Uniaxial tensile	Tensile strength Ultimate strain Breaking force	Indocyanine green staining and glutaraldehyde increase retina stiffness
Wollensak and Spoerl [27]	Pig		Retina, choroid	Uniaxial tensile	Tensile strength Young’s modulus	Choroid one order of magnitude stiffer than retina
Ugarte et al. [4]	Human	Ageing, AMD	Choroid	Inflation	Stress-strain relation Elasticity modulus	Age-related increase in choroid stiffness not exaggerated by AMD
Wollensak et al. [33]	Human		Retina	Uniaxial tensile	Tensile strength Ultimate strain Breaking force	ILM responsible for ~50% of retina tensile strength
Candiello et al. [59]	Chick, mouse	Ageing	ILM	AFM	Young’s modulus	ILM stiffness very similar for embryonic chick and neonatal mouse
Candiello et al. [54]	Human	Ageing	ILM	AFM	Young’s modulus	ILM becomes increasingly stiff with advancing age
Chen et al. [14]	Pig		Retina, choroid	Uniaxial tensile	Transition stress Transition strain Young’s modulus Anisotropy	Transition stresses and moduli of all layers lower at body temperature
Chen and Weiland [25]	Pig		Retina	Uniaxial tensile	Transition stress Transition strain Young’s modulus Transition modulus Anisotropy	Blood vessels contribute significantly to retinal stiffness
Franze et al. [56]	Guinea pig		Retina	AFM	Young’s modulus Anisotropy	Significant differences in regional retina stiffness
Chen and Weiland [31]	Pig		Retina	Uniaxial tensile	Transition stress Transition strain Young’s modulus Transition modulus	Retina stiffness lower at 26 °C when compared to stiffness at 37 °C
Henrich et al. [55]	Human		ILM	AFM	Young’s modulus Anisotropy	ILM stiffness lower on vitreal side compared to retinal side
Shahbazi et al. [2]	Human		Retina, choroid	US	Young’s modulus	Significant difference between healthy and AMD retina stiffness
Haritoglou et al. [57]	Human		ILM	AFM	Young’s modulus Anisotropy	Staining with vital dyes significantly increases the stiffness of the ILM
To et al. [60]	Human	Diabetes	ILM	AFM	Young’s modulus	ILM stiffness increased in diabetes
Chen et al. [7]	Human	AMD	Retina, choroid	Uniaxial tensile	Transition stress Transition strain Young’s modulus Toe modulus Heel modulus Anisotropy	Retina is anisotropic and properties of each layer may change with age
Chen and Weiland [34]	Human, pig	Ageing, AMD	Retina	Uniaxial tensile	Transition strain Young’s modulus Toe modulus Heel modulus	Increased retina stiffness in moderate-to severe AMD
Worthington et al. [37]	Mouse, pig	Ageing, Inherited retinal degeneration	Retina, choroid	Compression	Young’s modulus Anisotropy	Compressive modulus remains relatively stable with age
Pekel et al. [62]	Human	myopia	Retina, choroid	USE	Young’s modulus	Retina–Choroid–Sclera stiffness increases as axial length increases
Pekel et al. [63]	Human		Retina, choroid	USE	Young’s modulus	Photocoagulation increases stiffness of retina–choroid–sclera complex
Qian et al. [53]	Cat		Retina, choroid	OCT, IFE	Young’s modulus	Elastic modulus of choroid one order of magnitude higher than retina
Agladioglu et al. [64]	Human	POAG	Retina, choroid	USE	Young’s modulus	Retina–Choroid-Sclera stiffness similar in healthy and glaucomatous eyes
Vielmuth et al. [58]	Human		ILM	AFM	Young’s modulus Anisotropy	Similar ILM stiffness in eyes with or without ocriplasmin treatment
Qu et al. [44]	Pig	Induced retinal damage	Retina	ARF-OCE	Young’s modulus Anisotropy	Retinal stiffness increases from ganglion cells side to photoreceptors side
Qu et al. [71]	Rabbit		Retina	ARF-OCE	Young’s modulus Anisotropy	Retinal stiffness increases from ganglion cells side to photoreceptors side
Wang et al. [65]	Pig		Choroid	Uniaxial tensile, inflation	Young’s modulus	Bruch’s membrane may influence on IOP-induced ONH deformations
He et al. [45]	Pig, rabbit		Retina	ARF-OCE	Young’s modulus Anisotropy	Retinal stiffness increases from ganglion cells side to photoreceptors side
Ciasca et al. [28]	Human	MH/ERM	ILM	AFM	Young’s modulus Anisotropy	ILM retinal side significantly stiffer in MH than in ERM
Djigo et al. [36]	Human		Choroid	Uniaxial tensile	Tensile strength Ultimate strain Young’s modulus	Choroidal tensile strength of ~300 kPa

*AFM* atomic force microscopy, *AMD* age-related macular degeneration, *ARF-OCE* acoustic radiation force optical coherence elastography, *ERM* epiretinal membrane, *IFE* inverse finite element modelling, *ILM* internal limiting membrane, *IOP* intraocular pressure, *MH* macular hole, *OCT* optical coherence tomography, *ONH* optic nerve head, *POAG* primary open-angle glaucoma, *US* ultrasound, *USE* ultrasound elastography.

### Techniques used to assess retinal and choroidal biomechanics

**Fig. 1:**
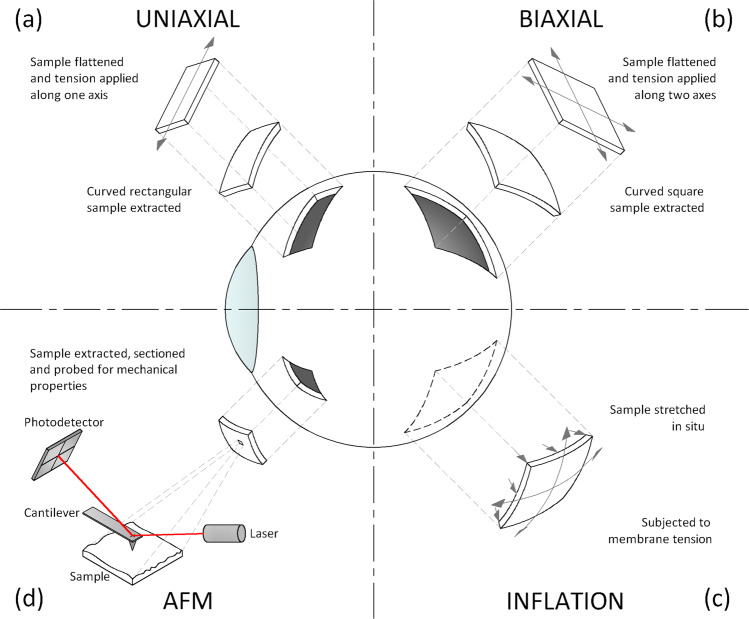
Biomechanical in vitro test methods. Schematic illustration of deformation modes experienced in commonly used test methods to characterise the mechanical properties of ocular tissues showing **a** uniaxial tension, **b** biaxial tension, **c** inflation and **d** atomic force microscopy.

Biomechanical properties of tissues are assessed by measuring the deformation in response to an applied force induced by different methods. Schematic illustrations of the tissue deformation modes experienced commonly used in vitro test methods are presented in Fig. 1.

### In vitro testing

Biomechanical tests to determine the mechanical properties of individual ocular tissues have traditionally been performed in vitro using ex vivo tissue postmortem, due to the difficulties in assessing them in vivo [6]. Eyes from various animal species including pigs, rabbits, primates and mice have all been used in biomechanical studies [14–18]; however, porcine tissue has more frequently been used due to its widespread availability, similarities in size, absence of a tapetum and holangiotic vascular pattern similar to primates. Indeed, Chen et al. evaluated the elastic properties of both porcine and human posterior eyewall, observing that each porcine tissue layer had elastic moduli within an order of magnitude of the values obtained from human tissues, and could be used as an eqivalent [7].

Variations in experimental setup can have a significant impact on the results obtained, as well as the direction and rate of stretch during an experiment due to the anisotropic and viscoelastic behaviour of ocular tissues, meaning that straightforward comparison of results from different studies is rarely possible [7, 14, 19–28]. Another concern with testing ex vivo tissue is post-mortem changes [6]. The processing of tissue samples is not standardised and several factors, such as the post-mortem processing time, as well as storage and transport conditions, can influence the measurements, limiting the reproducibility of the experiments [29]. Indeed, environmental factors, such as calcium concentration, pH, and temperature strongly affect the adhesion strength between the neurosensory retina and the retinal pigment epithelium (RPE) in the postmortem period [30]. Therefore, Chen et al. highlighted that in vitro conditions should match as closely as possible in vivo conditions [14, 25, 31]. For instance, both choroid and retinal stiffness were found to decrease with a temperature rise from 25 to 37 °C in 6-month-old porcine eyes [14]. Based on these findings, a 37 °C saline solution has been proposed as optimum for in vitro biomechanical testing to simulate physiologic conditions [7]. In contrast, it has been demonstrated that the freeze–thawing of BMCC does not apparently alter the tissue biomechanical properties in terms of elasticity and stress-strain relation [6]. Finally, the tissue may behave differently when in its normal anatomical relationship with other tissue, although there has been no quantification of this effect.

#### Uniaxial testing

Uniaxial tensile testing is currently the most common method used to determine retinal and choroidal biomechanics [7, 14, 18–25, 27, 31–37]. For tensile testing, the targeted tissue (e.g. BMCC, or retina with or without ILM) is dissected from enucleated eyes and strips of uniform width and desired length are excised. Each strip is then mounted between two grips/clamps and stretched in one direction (i.e. uniaxially) while recording the resulting load and elongation. While strip thickness has been measured using an ultrasound (US) pachymeter [38] or micrometre [21] for cornea and sclera uniaxial experiments, optical coherence tomography (OCT) [18] and histological methods [7, 14, 25, 27, 31, 33, 34, 36] have been used for the retina and choroid, as the pressure exerted using a micrometre could damage the tissue [21]. When the strip dimensions are considered, the stress and strain can be determined and, from this, further parameters. The strain, *ϵ*, is the response of the material to an applied force and is defined as:

Eq. 1 $${\it{\epsilon }} \,=\, \frac{{\Delta L}}{{L_0}}$$

where *ΔL* is the change in length and *L*_*0*_ is the original length of the strip.

The stress, *σ*, is the force per unit area generated within the cross-section of the material due to the strain and is defined as:

Eq. 2 $$\sigma \,=\, \frac{F}{A}$$

where *F* is the applied force and the *A* is the cross-sectional area of the strip.

**Fig. 2:**
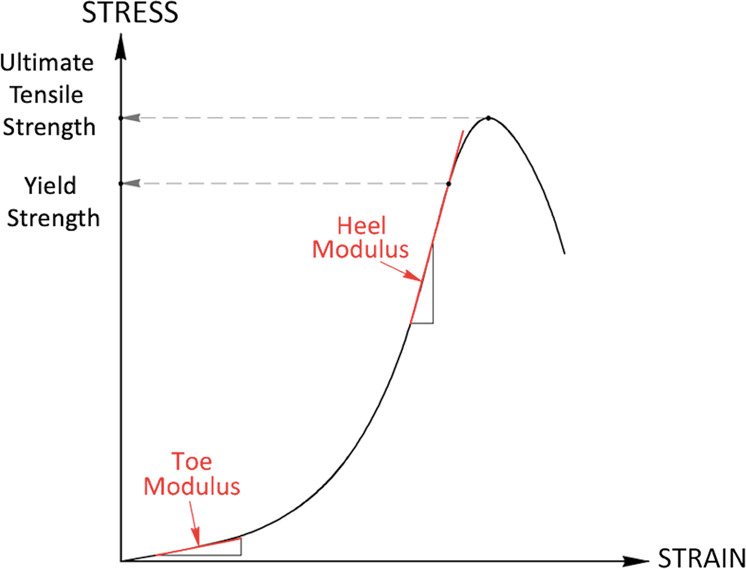
Stress–strain curve for soft tissues. Schematic illustration of a typical non-linear stress-strain curve for soft biological tissue showing the points at which the toe modulus, heel modulus, yield strength and ultimate yield strength are calculated.

The stress-strain behaviour of soft biological tissues typically follows a J-shaped exponential curve, as is reported in most studies for both choroid and retina (Fig. 2). In such a curve, the three regions identified have been attributed to the degree of collagen recruitment as the tissue is stretched: first, a plateau region in which the sample is not yet fully straightened; second, the elastic region, characterised by an increase in mechanical resistance due to the progressive recruitment of collagen fibres; [39] third, a region of “rupture” in which the mechanical resistance decreases and the sample starts to tear [14]. Upon unloading, energy dissipation within the tissue results in a lag behaviour which is evident in the difference between the loading and unloading stress-strain curves. When loaded to failure, the yield strength and ultimate tensile strength can also be quantified from the stress-strain graph as shown in Fig. 2.

The modulus of elasticity is a measure of a materials resistance to deformation within its elastic limit. For a linear elastic material, the change in stress is directly proportional to the change in strain and the modulus of elasticity remains constant. In this situation the parameter is referred to as the Young’s modulus, *E*, where:

Eq. 3 $$E \,=\, \frac{\sigma }{{\it{\epsilon }}}$$

However, the behaviour of soft biological tissues, such as the retina and choroid is non-linear, and the stress typically increases exponentially in relation to strain. Therefore, it is more appropriate to use the term tangent modulus, *E-tan*, as the value will be determined as the slope of a tangent line at a chosen point on the stress-strain curve. Tangent moduli are typically compared at specified physiologically relevant levels of stress [35] or strain [18]. Alternatively, the toe and heel modulus correspond to *E-tan* measured in the lower and upper slopes of the stress-strain curve, respectively, within the elastic limit [7, 34]. Higher values correspond to stiffer, less extensible material responses to stretching.

Uniaxial tests have been used to assess the effect of various factors on the biomechanical properties of the retina and choroid, namely ageing [7, 21, 23, 35], disease [34], strain rate [20, 27] and temperature[14, 19, 31]. Although restricted to assessing the localised uniaxial behaviour of the excised tissue strip, this method has also been used to investigate anisotropy, which is directly related to the orientation of collagen fibres within the tissue [40]. In the posterior eye, the anatomical landmark for sample harvesting is usually the ONH or the foveal centre, with strips excised in the horizontal or vertical direction [7, 14]. Less commonly, with circumferential (equatorial) or meridional (anterior to posterior) orientations for comparisons between the anterior [21], equatorial [23] and posterior regions, as well as regions with and without blood vessels have been used [20, 22, 24, 25]. Since retina and choroid collagen fibres are mainly aligned tangentially to the ocular surface, both tissues exhibit their maximum strength along surface directions with surface anisotropy [14, 22]. In this regard, biaxial tensile testing can be used to simultaneously extend the tissue equally along two axes, although only used to date for the sclera [41].

Despite the extensive use due to its simple setup and post-test analysis, uniaxial tensile has a number of limitations. Firstly, excising the tissue strip from its native curved environment results in severing of load-bearing collagen fibres on both sides along the length of the strip. Secondly, flattening of the strip can generate compression towards the outer surface and tension towards the inner surface, altering the measured biomechanical properties. Thirdly, applying tension along one axis is not representative of that which is experienced in vivo. Finally, cyclic preconditioning of the tissue is required to obtain reproducible behaviour as repeated cyclical straining from 0% to a pre-set maximum value of stress or strain at a predefined rate induces reorientation of elastin and collagen fibres toward the loading axis [6, 18, 42]; however, a stiffening effect has been related to preconditioning for sclera and cornea.

#### Inflation testing

Inflation testing has primarily been used for the biomechanical evaluation of cornea and scleral tissue [6, 42, 43], but also of the choroid [4, 18] and retina [44, 45]. The technique is widely regarded as the most desirable in vitro biomechanical method, due to its similarities to the in vivo situation whereby the pressurised eye is subjected to membrane tension. Enucleated eyes, typically transected or whole globes, are inflated using phosphate-buffered saline (PBS) to vary intraocular pressure (IOP) while monitoring the pressure change and tissue deformation. Preconditioning cycles are normally required to obtain repeatable behaviour from the tissue. In order to monitor tissue displacement (deformation) in situ, Ugarte et al. used OCT for human BMCC during inflation testing [4]. In particular, they mounted 4-mm diameter sections of isolated BMCC in a pressurisation chamber applying a positive pressure to the choroidal surface [4]. Cross-sectional OCT images were then used to calculate the arc length of the inflated membrane and from this the change in arc length due to applied pressure used to quantify the strain, *ϵ*, using Eq. 1. Rather than calculating cross-sectional area of the tissue to determine stress, *σ*, the applied pressure within the inflated membrane was considered to be the induced stress within the sample. Contrary to Eq. 3, the authors stated that the elastic modulus, *E*, was calculated as the ratio of strain to stress rather than stress to strain. Nevertheless, the determined values were used to assess the effects of freeze–thawing on the biomechanical properties of BMCC as well as age- and AMD-dependent variations. More recently, Wang et al. [18] used inflation testing to assess porcine BMCC rupture in conditions of elevated IOP. After removing a 7 × 7 mm square section of the overlying sclera, whole eye globes were progressively inflated using PBS until vitreous leakage through the exposed BMCC was evident. The pressure at this point was then registered as the value required to rupture the BMCC.

However, determining accurate material properties using inflation tests is demanding due to the varying thickness and anisotropic behaviour of ocular tissues.

#### Finite and inverse finite element modelling

Finite element (FE) modelling is a computer-aided numerical technique. The geometry of the structure, in this case the eye, is discretised into a mesh of individual or “finite” elements. By assigning representative boundary conditions and material behaviour characteristics to the elements, and incrementally simulating an applied load (e.g. IOP), the equations required to determine the resulting deformation of each element are solved computationally to obtain global deformation of the structure. Using this technique, valuable insights have been gained into trauma-induced retinal haemorrhage [46] and macular hole (MH) formation [47], ONH deformations [18], as well as vitrectomy-induced retinal shear stress [48] and optimal intravitreal injection angles [49].

For FE modelling, it is important to accurately define the geometry and material properties of the structure. However, due to its anisotropy, complex geometry and varying thickness, inverse FE analysis is often used to determine material properties of the eye. Inverse FE analysis is a combined experimental and computational process where experimental deformations are first monitored while the tissue is stretched due to an applied force. An FE model of the test specimen is then constructed and the material properties of its elements are optimised using an iterative process until the displacements observed during the experiment match those produced by the FE model.

The majority of inverse FE studies on ocular biomechanics have focused on the corneoscleral tunic in ex vivo models [50–52]. However, Qian et al. [53] applied inverse FE analysis to assess biomechanical properties of the choroid and retina in vivo. The authors injected saline into the anterior chamber of a cat eye while monitoring pressure using a pressure transducer. During the experiment, the choroid and retina were imaged using OCT at increasing levels of IOP, and the distance between locations in and around the ONH was used as control points to monitor deformations. However, the invasiveness of the approach used by Qian et al. would preclude its use in human participants.

#### Atomic force microscopy

Atomic force microscopy (AFM) is a nanoscale surface imaging technique, capable of assessing mechanical properties of biological materials. Small sections of tissue, typically a few mm^2^ in size, are removed from their native environment and mounted in the AFM on glass slides. The AFM has a cantilever with a tip, also known as a probe, which scans the sample surface. In order to register the vertical and lateral motion of the probe during scanning, a laser beam is reflected off the cantilever and tracked through a deflection sensitive photodetector. Knowing the cantilever stiffness, the Young’s modulus of the samples can be calculated from the deflection of the cantilever as it indents points on the sample surface. AFM has been used to assess thickness [54, 55] and a range of ILM biomechanical parameters, namely anisotropy [28, 55–58], indentation rate sensitivity and hysteresis [28], developmental and age-dependent stiffness variations [54, 59], as well as stiffness changes due to disease [28, 60] and treatments, such as tissue staining [57, 58] and glycosaminoglycan removal [54]. While AFM is restricted to scanning small areas in the region of 150 × 150 × 20 µm, the primary advantage of this technique is its ability to measure properties of nanoscale structures such as cells, collagen and nerve fibres. However, several variables must be carefully considered: firstly, the cantilever stiffness should be similar to the stiffness of the sample being tested; secondly, the substrate on which the sample is mounted can influence the results; thirdly, the most commonly used mathematical model (i.e. the Hertz model [61]) assumes linear elastic behaviour and homogeneity of the sample, and so it is important to focus on the region of the probe force-deflection curve which represents the structure that is being investigated.

### In vivo testing

In vivo measurements of biomechanical properties of ocular tissues by non-invasive techniques may lead to a patient-specific assessment of both risk factors for the development and progression of ocular diseases and prognostic factors related to surgical treatment. Limited data are currently available regarding the use of high-resolution magnetic resonance imaging (MRI), ultrasound biomicroscopy and OCT. MRI has been used to assess sclera and for the evaluation of IOP-induced changes of the whole globe with particular regard to the posterior sclera displacement [17]. However, random eye movements in living or non-paralysed subjects can lead to blur artefacts and MRI has never been used to assess thinner tissue, such as retina and choroid [17].

#### Ultrasound elastography

Ultrasound elastography has been proposed as a reproducible method to assess the in vivo elasticity of the retina–choroid-sclera complex in several pathologic conditions. An ultrasonic transducer is used to transmit inaudible, high-frequency soundwaves into the eye while the response is sensed by a second transducer which can, in some cases, be the sending transducer. Pekel et al. used US elastography to assess elasticity in myopic [62] and diabetic eyes treated with argon laser panretinal photocoagulation [63]. After applying gel, the ultrasound probe was placed in contact with the closed eyelid and small rhythmic compressions were manually applied by the operator. A similar approach was used by Agladioglu et al. [64] to assess ocular elasticity patients with primary open-angle glaucoma. Tissue elasticity in these studies was determined by the ultrasound system which provided unitless values that can be used to distinguish between areas of high and low stiffness. Shahbazi et al. [2] used US to determine quantitative biomechanical parameters of the retina–choroid complex in healthy and AMD patients. In this study, the US probe monitored static pressure-induced changes in axial length and tissue thickness from which strain and elastic modulus values were then estimated [2]. However, this technique has been not yet been used to individually characterise properties of the retina and choroid.

#### Optical coherence elastography

Optical coherence elastography (OCE) combining structural OCT imaging with elasticity measurement principles is able to provide tissue elasticity mapping with high sensitivity and high spatial resolution (about 10 µm) [65–69]. In this technique, the OCT detects the deformation or vibration of the sample induced by an external force that can be generated by different techniques, such as air-puff pulse [65], acoustic radiation force (ARF) [66], needle probe [67], piezoelectric transducer [68] and laser pulse [69]. The excitation of the sample generates elastic waves propagating to the surrounding tissues. The ability to measure wave velocity and track wave propagation by the OCT provides a means of quantifying and mapping the elastic properties. Bulk moduli for longitudinal wave propagation are based mainly on water, and is dependent on short-range molecular interactions, while shear moduli are more related to tissue structure [70]. Consequently, bulk moduli values for biological tissues fall within approximately one order of magnitude, whereas shear moduli range over several orders of magnitude. Therefore, monitoring shear waves provides greater contrast between different tissue types and increases the potential for distinguishing between healthy and diseased tissues [70].

Although ARF is able to excite both superficial ocular tissue, such as cornea, and deeper tissue, such as retina and choroid, the use of elastography methods to assess retinal and choroidal biomechanics has been limited due to the inaccessibility of such tissues and unsatisfactory resolution [44]. However, ARF-OCE has recently been used to evaluate the in vivo elastic modulus of the choroid and individual retinal layers in rabbit eyes [45, 71]. While Qu et al. [71] acknowledged that the acoustic intensity used to excite the ocular tissue in their study exceeded FDA limits for diagnostic ultrasonography of the human eye, the required intensity reduction to satisfy the guidelines would still induce detectable tissue displacements. However, the current methodology required proptosis to expose the sclera during measurements and so, to quantify posterior eye biomechanics in vivo, further refinement is required to translate the technology to a clinical setting.

## Retina

**Fig. 3:**
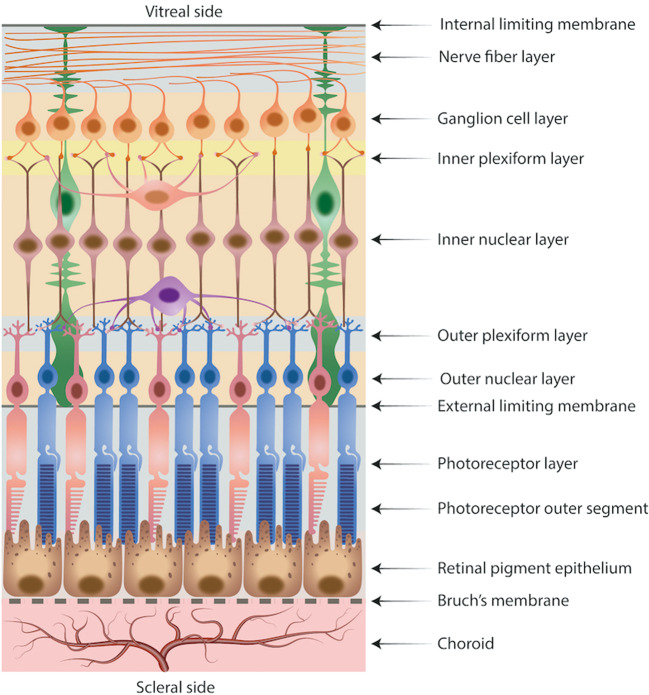
Retina and choroid. Schematic diagram of retinal layers, Bruch’s membrane and choroid.

The retina, the innermost tissue of the eye, is composed of neuronal and non-neuronal cells organised in laminated structure with a thickness in humans of around 250 µm [3]. In particular, the neurosensory retina is composed of the nine distinct layers from the internal limiting membrane on the vitreous side to the photoreceptor layer (Fig. 3). The neurosensory retina, hereon called the retina, rests upon a specialised monolayer of hexagonal cells joined by tight junctions and forming the outer blood retinal barrier, the RPE, having crucial functions for the maintenance of retinal health [72]. The mechanical inter-digitation of the RPE microvilli to the outer segments of the photoreceptors, the maintenance of subretinal ions concentrations and the transport of subretinal fluid towards the choriocapillaris are all mechanisms that actively contribute to the adhesion between the RPE and neurosensory retina. The role of Müller cells for the homoeostasis and the structural integrity of the neurosensory retina is also crucial [73]. These specialised radial glial cells have two main stem processes radiating from the body in two opposite directions, one towards the vitreal surface and the other towards the photoreceptors, and ending in the ILM and ELM, respectively [73].

The distribution, density and morphology of the above cells vary across the retina topographically but also with age or disease [74]. These spatial variations in retinal cells distribution and arrangement could contribute to topographical variations in retinal stiffness [73]. Using AFM in guinea pig eyes, Franze et al. demonstrated that retinal stiffness increased moving outwards from the ONH to a distance of 2.5 mm, remained stable in the midperiphery and, then decreased in the far periphery [56]. Moreover, the stiffness of temporal and nasal retinal quadrants was significantly higher than that of the inferior and superior quadrants [56]. However, in the retina, as well as in the choroid, it is thought that elastin and collagen are mainly responsible for the tissue’s stiffness, whilst proteoglycan molecules, mainly heparan and chondroitin sulphate proteoglycans, contribute to its incompressibility [75]. A reduction of retinal elastin and, consequently, increased retinal stiffness has been demonstrated in patients affected by moderate-to-severe AMD [34]. It is known that a variety of tissue insults result in an increased production of proteoglycans [75, 76]. Consistently, a significantly higher content of proteoglycans has been reported in maculae with early AMD. Moreover, heparan sulphate proteoglycans (HSPGs) also appear to be involved in the pathogenesis of AMD, due to their reduced ability to bind complement factor H and, as a result, regulate the alternate pathway of complement, whose inappropriate activation plays a critical role in the progression of AMD [75].

From a mechanical point of view, the first investigations of the retina with regard to its elastic behaviour estimated Young’s modulus to be ~20 kPa [77]; this value was determined using uniaxial testing on isolated bovine retina. The retina however is an anisotropic and heterogeneous tissue, with its non-linear elastic behaviour differing between the vertical and horizontal meridians [7]. Indeed, Chen et al. using uniaxial testing on human retinal strips, showed that retinal transition stress, heel modulus and toe modulus all tended to be higher in the vertical, compared to the horizontal direction [7]. The authors reported that the retinal heel modulus was ~19 and 13 kPa in the vertical and horizontal meridians, respectively, which is of translational relevance when peeling adherent membranes from the retinal surface to avoid retinal tearing. Moreover, the retinal stress-strain curve showed that, above an inflection point, the stiffness decreased as the strain increased [7]. Anisotropic differences in retinal tensile responses have also been described for the meridional (i.e. axially, anterior to posterior) and equatorial (circumferential) axes, as retinal strips in the former orientation endure higher stresses than the latter [24]. In addition, blood vessels significantly contribute to retinal stiffness [25].

The mechanical properties have been also studied in relation to ageing. Reinchebach et al. demonstrated that in adult rabbit eyes all retinal regions showed reduced tensility (i.e. reduced ability to extend) when compared with neonatal tissue, consistent with a progressive increase in retinal elasticity during development [78]. However, the retinal compressive modulus appears to be relatively stable with age [7]. Retinal strain anisotropy appeared to decrease with age due to the decrease of the vertical transition strain and the increase of horizontal transition strain [7]. However, the authors, constrained by tissue access, only analysed 24 eyes with a strong male predominance and a minimum age of 30 years. Moreover, one of the main limitations of in vitro retinal strip testing lies in the isolation of the retinal samples itself. In vivo, the neurosensory retina is strongly adherent to the RPE, which adds to its biomechanical strength and properties. Such adhesive force has been studied in living eyes of different species and assessed at 0.001 N/cm on average in rabbits [79]. Adhesion is stronger in cats and monkeys, which show mean values that are 180% (for cats) and 140% (for monkeys) of that in rabbits [79].

Recently, Qu et al. used ARF-OCE to measure the elasticity of the retinal layers ex vivo in porcine eyes [44]. The authors found that the stiffness decreased from the top to the bottom layers, with Young’s modulus increasing from 1.3 kPa in the inner retinal layers to 26 kPa in the photoreceptor layer, consistent with the proximity of the photoreceptor layer to the choroid and sclera whose stiffness is much higher than the retina [44].

## Internal limiting membrane

The ILM is formed principally by the basement membrane of the Müller cells, forming the inner surface of the retina [80]. Structurally, the main components are collagen type IV, fibronectin and laminin. The thickness varies from 0.01 to 0.10 µm at the optic disc and the fovea, to 0.5–3.2 µm at the posterior retina [80]. The posterior vitreous cortex is firmly and broadly adherent to the vitreal side of the ILM through adhesion molecules, including fibronectin, laminin, and heparan sulphate proteoglycans, forming the VRI [80]. With age, vitreous liquefies and the gel structure collapses. Combined with this, there is a weakening of the VRI, and the vitreous separates from the ILM in a stereotypical way, peri-foveally initially, followed by the fovea and then optic disc. In vitreoretinal surgery, ILM removal is commonly peeled off for a variety of indications by using end-gripping forceps to remove tangential traction at the retinal surface. Since the ILM is thin and transparent, different dyes have been commonly used to improve ILM visualisation and minimise surgical trauma [81].

The biomechanical properties of ILM have been investigated using the AFM [55, 57, 60]. In most studies, after dissection of fragments of chick, mice or human cadaveric retina, the ILM has been isolated by incubation in 2% Triton-X-100, as basement membranes are detergent-insoluble [54, 60]. The ILM is mechanically stronger and stiffer than the cellular layers of the retina being 1000-fold stiffer, and accounting for ~50% of retinal tensile strength. This is comparable to articular cartilage, suggesting it has a major role in the structural integrity of the retina [55, 59]. Moreover, its retinal side, where there is a higher density of proteins typical of extracellular matrix [55], is over five times more rigid than the vitreal side [55, 57]. Evaluating human ILM in 2-mm^2^ retinal segments taken from within the vascular arcades, Henrich et al. [55] assessed the variation in ILM thickness and stiffness from the foveal centre. The ILM thickness and stiffness which were closely related, reached their maximum at about 1000 µm from the foveal centre, then decreased progressively towards the periphery, which can help guide surgical approaches for peeling [55]. Candiello et al. [54] demonstrated that the thickness and the stiffness of human ILM, outside the posterior pole, increase in an age-dependent manner. It has to be noted that, as the ILM acts as a barrier against the access of therapeutic antibodies, viruses or cDNAs injected intravitreally, differences in thickness are likely to have a role on the effect of these agents on retinal targets [82].

As already observed, ILM plays an important role in several vitreoretinal diseases. Ciasca et al. [28] used AFM to compare ILM specimens obtained from patients who had undergone PPV and ILM peeling due to MH and epiretinal membrane (ERM). They found that the ILM retinal side was significantly stiffer in cases with MH than in ERM, suggesting that, in the former, the higher ILM stiffness could stabilise and strengthen the adhesion between ILM and Müller cells [28].

As ILM peeling can be a challenging technique, various vital dyes have been introduced as intraoperative tools to facilitate ILM visualisation for peeling [57]. It has been demonstrated that different dyes not only exhibit different interactions with surrogate ILM membrane models [83] but also result in different histological planes of separation of the peeled ILM from the underlying retina [84] and different immunohistochemical findings of the peeled ILM [85]. To evaluate the potential influence of vital dyes on the biomechanical properties of human ILM, Haritoglou et al. [57] collected unstained ILMs peeled from human eyes undergoing vitreoretinal surgery and analysed them with AFM. Samples were stained with brilliant blue G (BBG) 0.025% or indocyanine green (ICG) 0.05% and an unstained fragment was used as a control [57]. They also illuminated the stained fragments for 1 min with a standard vitreoretinal light source to assess any variations related to intraoperative illumination [57]. The authors reported that the rigidity of ILMs on both the retinal and vitreal sides significantly increased after staining with ICG and BBG [57]. The increased stiffness was more pronounced on the vitreal side and with ICG [57]. In addition, there was a further increase in stiffness, of around 1.2-fold, with ICG after illumination, but not with BBG [57].

Diabetes has been associated with changes in the composition of ILM with resultant changes in its biomechanical properties [60]. Long-standing diabetes is characterised by significant thickening of basement membranes with the formation of advanced glycation end products (AGEs) of their constituent proteins [86]. Several extracellular matrix components have been shown to be increased in diabetic patients, namely fibronectin (on the retinal side), laminins, collagen types I, III, IV and V, and heparan sulphate proteoglycans [60, 87]. Using AFM on ILM obtained from cadaveric human eyes, samples from diabetic donors were stiffer than those from age-matched non-diabetic donors, with the differences ranging from 20 to 60%, and explaining perhaps the observed variability in ILM characteristics noted during surgical peeling in diabetic retinopathy [60]. Indeed, it has been reported that the ILM peeling in diabetic patients may result in more severe damage due to predisposing alterations of diabetic ILM, such as the presence of proliferating cells resulting in increased thickness and stronger adhesion between vitreous cortex and ILM due to both crosslinking of collagen fibrils and AGEs and the further activation of Müller cells induced by hyperglycemic condition [88].

However, it is worth noting that AFM only has limited penetration of tissue samples and, therefore, provides measurements relative to the tissue surface. Moreover, changes in ILM in association with gender and other posterior segment diseases have not yet been studied.

## Bruch’s membrane/choroid complex (BMCC)

Bruch’s membrane is a 2–4 μm-thick acellular sheet positioned between the RPE and the choroid. It is composed of five distinct layers [89]. The main components of BM are collagen types I, III, IV, V and VI, fibronectin, laminin, elastin and proteoglycans, in particular heparin and chondroitin sulphate proteoglycans [89]. Bruch’s membrane acts as a physical and biochemical barrier for both molecules and cells between the retina/RPE and the choroid, as reservoir of anti-angiogenic factors and as scaffold for the adhesion, growth and support of the RPE cells [89, 90].

It has been suggested that changes in the biomechanical properties of BM are associated with AMD [91]. It is known that BM undergoes several changes with age, including an increase in thickness with a blurring of the boundaries between the five layers, accumulation of lipids as well as AGEs and a decrease in both the amount and sulfation of heparin sulphate proteoglycans in the macular area [89, 92]. Moreover, a physical breakdown of BM has been associated with both aging and AMD [89]. The involvement of BM has been also hypothesised in the physiological process of emmetropisation as well as pathological myopisation, through the generation of an active force in the midperiphery, influencing the axial elongation of the globe [93]. Since it is difficult to isolate the BM, the biomechanical properties of this tissue have been commonly indirectly assessed through the biomechanical study of the BMCC [4, 18, 21].

The choroid is the 200 µm-thick vascular layer located between the sclera and the retina, responsible for the supply of oxygen and nutrients to the outer retina [6, 94]. Going from the retinal to the scleral side, the choroid is structured in several layers, namely: the choriocapillaris, a capillary network with maximum thickness of 10 μm at the fovea, progressively decreasing to about 7 μm peripherally; Sattler’s layer, composed of arterioles feeding the choriocapillaris and medium/small arteries and veins; Haller’s layer, composed of large blood vessels; the suprachoroid a transitional zone containing elements of both choroid and sclera, such as collagen fibres, melanocytes and fibroblasts; and, finally, the lamina fusca, a 30 μm-thick layer separating the suprachoroid from the sclera [6, 94]. In the choroid, the flow per perfused volume is the highest of any other human tissue, and the choroidal vessel and capillaries contain about 85% of overall ocular blood flow [94]. Structurally, the main components of this tissue are heparan sulphate, laminins, collagens type IV, V, and VI, and a network of elastic fibrils [6, 95]. The elastic network is connected to both the posterior tendons of the ciliary muscle and a network of contractile cells extending from the optic nerve to the area of the vortex veins [96]. Through this connection, the contractile cells have been hypothesised to counteract the variations in diameter and position of the choroidal vessels potentially induced by the pulling action of the ciliary muscle towards the elastic network during accommodation [95, 96]. Moreover, the ability of the choroid to modulate its thickness is also thought to be important for emmetropisation [97]. It is also worth noting that changes in choroidal volume and, consequently, thickness result in changes of IOP [98].

To date, the literature on BMCC biomechanics has been limited as its influence on the stiffness of the eye has been supposed to be negligible [6]; however, there is a growing interest in their mechanical properties as their role in ocular development and pathologic conditions has become clearer.

Uniaxial tensile tests on choroidal strips have been used to investigate BMCC elasticity in relation to surgical procedures and trauma [7, 21]. The BMCC behaves as a non-linear soft tissue as the stiffness increases with stretching [18]. Unlikely the retina, human choroid shows significantly higher stiffness and no significant difference in elastic behaviour between the vertical and horizontal meridians (Heel modulus of 387 and 362 kPa for choroid vs. 19 and 13 kPa for retina in the vertical and horizontal meridians, respectively) [7]. However, it has been reported, similar to retina that meridional choroidal strips are stiffer than equatorial ones [19]. Moreover, the elastic modulus of BMCC-RPE complex strips was significantly greater in samples taken posterior to the equator than those taken more anteriorly [21], whereas the elastic modulus of radial choroidal strips (straddling the equator) did not significantly change by location (superior, inferior, temporal or inferior) [21].

Compared with scleral tissue, human specimens of BMCC exhibit lower stiffness e.g. mean Heel modulus ~370 kPa versus 4400 kPa and a more linear stress-strain curve [7, 21]. On the contrary, testing BMCC specimens excised from porcine eyes, Wang et al. [18] found that BMCC samples had elastic moduli (~1–2 MPa) at least comparable or higher than those reported for sclera (~1–8 MPa) [99], and far higher than the retina, cornea and iris (~0.01, 0.3 and 0.004 MPa, respectively) [99, 100]. The authors argued that their results could be due to the smaller amount of choroid included in the specimens compared with previous studies and, therefore, could be more representative of BM biomechanical properties rather than BMCC [18]. Using inflation tests on completely excised BMCC specimens and assessing their deformation with OCT, Ugarte et al. reported that BMCC stiffness significantly increases with age, potentially leading to a reduction of choroidal blood flow with consequent alterations to the oxygen and nutrient supply to the retina [4]. Finally, it has been reported that a substantially high IOP can be sustained by BMCC alone, that in burst tests exhibited a rupture pressure of about 80 mmHg, with significant deformation before reaching the point of mechanical failure [18].

Graebel and van Alphen suggested a tendency for choroidal elasticity to decrease with age [35] and an increase in the horizontal stiffness of human choroid with age has been recently demonstrated using uniaxial testing [7]. Moreover, the decreased elasticity of BMCC with age does not appear to be exaggerated in AMD, as demonstrated by comparing BMCC samples taken from human donor eyes with and without signs of AMD, although further study is needed [7].

## Conclusion and future perspectives

The study of ocular biomechanics is a research area of growing interest due to its significant translational value with both diagnostic and therapeutic implications [10]. This aspect has been clearly highlighted for ocular tissues such as cornea and sclera, whereas retina and choroid have not been investigated so extensively so far. With regard to diagnosis, alterations in mechanical properties at cellular and tissue level are thought to determine the onset or progression of detectable structural alterations. For instance, a decrease of retinal elastin and, consequently an increase in stiffness, has been detected in moderate-to-severe AMD but not in early disease [37]. and there are other changes in the composition of Bruch’s membrane that are associated with both ageing and disease. Furthermore, although changes to the stroma will account for changes in the bulk mechanical properties of a tissue, the potential contribution of the direct contact point (the basement membrane for sheets of cells) has not yet been studied in a specific clinical entity but should be taken into consideration. In particular, each basement membrane contains two linked supramolecular networks of type IV collagens, and of laminins. Changes in crosslinking of type IV collagen, laminin isoforms, ratio of polymerising to non-polymerising laminins, concentrations of matrix remodelling proteins, such as matrix metalloproteinases, and post-translational modifications, such as glycosylation, can change the structure of the extacellular matrix and, thus, influence the biomechanical properties.

Knowledge of the biomechanical properties of ocular tissues is crucial to optimise surgical techniques and devices. Biomechanics could help predict the response of targeted tissues to surgery and the remodelling of such tissues after surgical manoeuvres or the implantation of various medical devices [10]. One of the main aims therefore of the current research into biomechanics is the development and improvement of testing methodologies in vivo, for both the whole globe and the individual ocular layers. This could result in the use of biomechanical tests in clinical practice as well as allow the optimisation of FE models of the eye [10].

In conclusion, the study of retinal and choroidal biomechanics is worthy of further investigation with potential to improve both the diagnosis and therapy of a variety of sight-threatening diseases.

## Footnotes

1 *Anisotropy: property of materials whose mechanical properties change in different directions (this is explained in the section “uniaxial testing”). Elasticity: property of materials able to resume their original shape and size after the removal of forces inducing their deformation. Hyperelasticity: property of materials for which the stress-strain relationship is exponential. Viscoelasticity: property of materials exhibiting both elastic and viscous behavior in response to deformation.
